# Persistence, switching, and discontinuation patterns of b/tsDMARDs in patients with rheumatoid arthritis: a single-center retrospective cohort study from Saudi Arabia

**DOI:** 10.3389/fmed.2026.1855256

**Published:** 2026-07-24

**Authors:** Saad A. Aldosari, Areej Alkhaleel, Mukhtar Alomar, Laila Alghalawin, Ahmad Alamer

**Affiliations:** 1 Department of Clinical Pharmacy, College of Pharmacy, Prince Sattam Bin Abdulaziz University, Al-Kharj, Saudi Arabia; 2 Department of Pharmaceutical Affairs, Dammam Medical Complex, Dammam, Saudi Arabia

**Keywords:** biological therapies, drug survival, persistence, real-world evidence, rheumatoid arthritis, Saudi Arabia, targeted synthetic DMARDs

## Abstract

**Objective:**

To characterize real-world treatment patterns, including persistence, switching, and discontinuation, of biologic and targeted synthetic disease-modifying antirheumatic drugs (b/tsDMARDs) in patients with rheumatoid arthritis (RA) in Saudi Arabia.

**Methods:**

This retrospective cohort study was conducted at Dammam Medical Complex in Saudi Arabia. Adult RA patients naïve to b/tsDMARDs who initiated therapy between January 1, 2013, and January 31, 2023, were included. Treatment patterns were summarized using descriptive statistics. Drug survival was assessed with Kaplan–Meier curves and Cox proportional hazards models.

**Results:**

Among 175 eligible patients, first-line therapy was a tumor necrosis factor inhibitor (TNFI) in 109 (62.3%), a non-TNFI in 47 (26.9%), and tofacitinib in 19 (10.9%). Estimated drug survival probabilities at 5 years were 0.47 (95% CI: 0.37–0.59) for TNFIs, 0.51 (95% CI: 0.38–0.68) for non-TNFIs, and 0.68 (95% CI: 0.48–0.97) for tofacitinib. At 10 years, survival was 0.33 (95% CI: 0.23–0.48) for TNFIs and 0.27 (95% CI: 0.16–0.45) for non-TNFIs. Kaplan–Meier analysis showed no significant difference across classes (log-rank *p* = 0.68). Adjusted hazard ratios for switching or discontinuation (reference: TNFIs) were 1.10 (95% CI: 0.69–1.74, *p* = 0.68) for non-TNFIs and 0.68 (95% CI: 0.27–1.72, *p* = 0.42) for tofacitinib. The primary reason for switching was lack/loss of efficacy, while pregnancy and non-adherence were the most frequent causes of discontinuation.

**Conclusion:**

Tumor necrosis factor inhibitors were the most commonly initiated first-line b/tsDMARD in this Saudi cohort. Persistence appeared comparable across classes, although the small sample size limits definitive conclusions. These findings provide initial real-world insights into the utilization of advanced RA therapies at a single tertiary center in Saudi Arabia.

## Introduction

Rheumatoid arthritis (RA) is a chronic, progressive, inflammatory disease characterized by symmetrical joint pain and swelling, often accompanied by systemic manifestations that significantly impair physical function and quality of life (1). Recent estimates indicate that RA affected approximately 17.6 million people worldwide in 2020 (95% uncertainty interval [UI] 15.8–20.3), with an age-standardized prevalence of 208.8 cases per 100,000 population (95% UI 186.8–241.1)—representing a 14.1% increase (95% UI 12.7–15.4) since 1990 (2). In Saudi Arabia, the only direct population-based prevalence study on RA, conducted in the Qassim region in 1998, reported a point prevalence of 2.2 per 1,000 people (0.22%) (3). More recent regional data from the Middle East and North Africa (MENA), including Saudi Arabia, show a steady rise in the age-standardized burden of RA since 1990. By 2019, point prevalence in the MENA region had increased by 28.3% (95% UI 25.5–30.9) to 120.6 cases per 100,000 population (95% UI 107.0–135.7), and annual incidence had risen by 25.2% (95% UI 22.4–27.7) to 5.9 cases per 100,000 (95% UI 5.2–6.6). In Saudi Arabia specifically, the increases were particularly pronounced, with age-standardized prevalence rising by 43.5% (95% UI 37.5–50.1) and incidence by 42.3% (95% UI 36.9–48.6). These estimates are modeled using the Global Burden of Disease (GBD) 2019 inputs (systematic reviews, claims data, vital registration, etc.) via Bayesian meta-regression (DisMod-MR 2.1). However, direct country-specific population-based surveys remain limited (4).

Given the increasing burden of RA, timely and effective management is essential to mitigate long-term disability and improve patient outcomes. The primary goals of managing RA include preventing joint damage, halting disease progression, achieving remission or low disease activity, enhancing quality of life, and controlling inflammation (5). Disease-modifying antirheumatic drugs (DMARDs) are the mainstay of RA treatment, alleviating symptoms and delaying radiographic joint damage. DMARDs fall into three main categories: conventional synthetic DMARDs (csDMARDs), biologic DMARDs (bDMARDs), and targeted synthetic DMARDs (tsDMARDs). csDMARDs include methotrexate (MTX), leflunomide, hydroxychloroquine, and sulfasalazine. bDMARDs are subdivided into four main classes, including tumor necrosis factor inhibitors (TNFIs) (adalimumab, certolizumab pegol, etanercept, golimumab, and infliximab), T-cell costimulatory inhibitors (abatacept), IL-6 receptor inhibitors (tocilizumab, sarilumab), and anti-CD20 antibodies (rituximab). The newer tsDMARDs, which inhibit Janus kinase (JAK), include tofacitinib, baricitinib, and upadacitinib (6).

The key treatment principles are clearly described in the international guidelines issued by the Saudi Society of Rheumatology (SSR), the American College of Rheumatology (ACR), and the European Alliance of Associations for Rheumatology (EULAR) (7–9). All three guidelines endorse a treat-to-target (T2T) strategy, in which therapy is intensified or adjusted based on regular monitoring of disease activity until remission or low disease activity is achieved. Under this framework, csDMARDs—most commonly methotrexate—serve as first-line therapy. For patients with an inadequate response to csDMARDs, no single b/tsDMARD is universally preferred; selection is individualized according to comorbidities, safety considerations, cost, and patient preferences regarding formulation and administration.

Although randomized controlled trials (RCTs) have demonstrated the efficacy and safety of biologics in RA, observational studies and registries provide broader, real-world evidence on long-term outcomes, including effectiveness, safety, tolerability, and treatment patterns. With the expanding approval and availability of diverse biologic and targeted therapies, there is an increasing demand for real-world studies to evaluate persistence, switching, and discontinuation in routine clinical practice.

A recent systematic review and meta-analysis of 182,098 RA patients found lower effectiveness among TNFIs compared with non-TNFIs (RR: 0.88; 95% CI: 0.81–0.95; *p* < 0.01; *I*^2^ = 94.0%) and among bDMARDs compared with JAK inhibitors (RR: 0.86; 95% CI: 0.79–0.94; *p* < 0.01; *I*^2^ = 93.0%), with higher effectiveness for adalimumab, etanercept, and golimumab than for infliximab (RR: 1.19; 95% CI: 1.05–1.36; p < 0.01; *I*^2^ = 96.0%) (10). Similarly, a Taiwanese study of biologic-naïve RA patients initiating etanercept, adalimumab, golimumab, tocilizumab, abatacept, or tofacitinib reported highest drug survival for tocilizumab and lowest for adalimumab; compared with etanercept, only adalimumab showed a higher risk of switching/discontinuation (adjusted HR = 1.17; 95% CI: 1.08–1.28), while golimumab, non-TNFI bDMARDs, and tofacitinib were less likely to switch or discontinue (11).

Given limited data on RA treatment patterns in Saudi Arabia, we aimed to describe persistence, switching, and discontinuation among patients with RA who initiated bDMARDs/Janus kinase inhibitors (JAKI).

## Materials and methods

### Study design, setting, and subjects

We conducted a retrospective observational cohort study at the specialized rheumatology clinic of Dammam Medical Complex (DMC) in Dammam, Saudi Arabia. Adult patients aged ≥18 years who initiated treatment with b/tsDMARDs between January 1, 2013, and January 31, 2023, were included.

Patients were excluded if they met any of the following criteria: (1) age <18 years at the time of RA diagnosis or treatment initiation; (2) diagnosis of active or latent tuberculosis (TB); or (3) concomitant diagnosis of psoriasis, psoriatic arthritis (PsA), ankylosing spondylitis (AS), inflammatory bowel disease (IBD), Crohn’s disease (CD), systemic lupus erythematosus (SLE), Behcet disease, gouty arthritis, or sacroiliitis. Patients with active or latent TB were excluded due to the increased risk of TB reactivation with b/tsDMARDs, while those with the other listed concomitant conditions were excluded to ensure cohort homogeneity and minimize confounding in this RA-specific study.

### Study outcomes and definitions

Persistence was defined as continuous use of the index b/tsDMARD without switching or discontinuation until the end of follow-up, loss to follow-up, or death. Switching was defined as the initiation of a different b/tsDMARD within 90 days after the end of the previous prescription’s supply (including any remaining days supplied). Discontinuation was defined as a gap of more than 90 days following the end of the previous prescription’s supply without initiation of the same or another b/tsDMARD. Patients transferred to other medical centers were censored at the date of their last documented encounter or prescription at DMC and were not counted as discontinuers.

### Statistical analysis

All analyses were performed using R software, version 4.0.1 (35) (R Foundation for Statistical Computing, Vienna, Austria). Data were entered into the Research Electronic Data Capture (REDCap) system in a de-identified manner (12). Descriptive statistics were used for baseline characteristics. Continuous variables were reported as medians with interquartile ranges (IQRs), and categorical variables as frequencies and percentages.

Missing data were summarized descriptively. Rheumatoid factor (RF) status was not included in the Cox models due to the high proportion of missing data (60.6%). ACPA status was not collected because it is not routinely performed or documented at DMC and is therefore not reported in this retrospective study. Sensitivity analyses restricted to patients with known RF status could not be performed due to the substantial missingness in this variable.

For the treatment persistence analysis, we used a time-to-event approach with the Kaplan–Meier method. Time zero was defined as the date of initiation of the index bDMARD or tofacitinib, with the event defined as discontinuation or switching of the index therapy for any reason (whichever occurred first). Observations were censored at the time of loss to follow-up or at the end of the study period. Kaplan–Meier survival curves were stratified by biologic therapy class (the only covariate included).

As a non-parametric method, Kaplan–Meier estimation requires fewer assumptions than fully parametric survival models (e.g., no assumption about the shape of the hazard function) and does not require proportional hazards (thus, no test for parallelism of survival curves is needed). However, like most standard survival methods, it assumes non-informative censoring (i.e., censoring is independent of the event risk). To address temporal changes in clinical practice over the study period, we stratified the analysis into two periods: 2013 to 2018 and 2019 to 2023. These periods were selected based on our knowledge that tsDMARDs (i.e., tofacitinib) became available in 2019. The stratified analysis was performed using the log-rank test.

Cox proportional hazards regression models were used to identify factors associated with drug persistence, including age, body mass index (BMI), concomitant use of steroids and methotrexate, and comorbidity with diabetes mellitus.

No formal sample size calculation was performed due to the study’s descriptive and exploratory nature.

## Results

### Demographics and baseline characteristics

**Figure 1:**
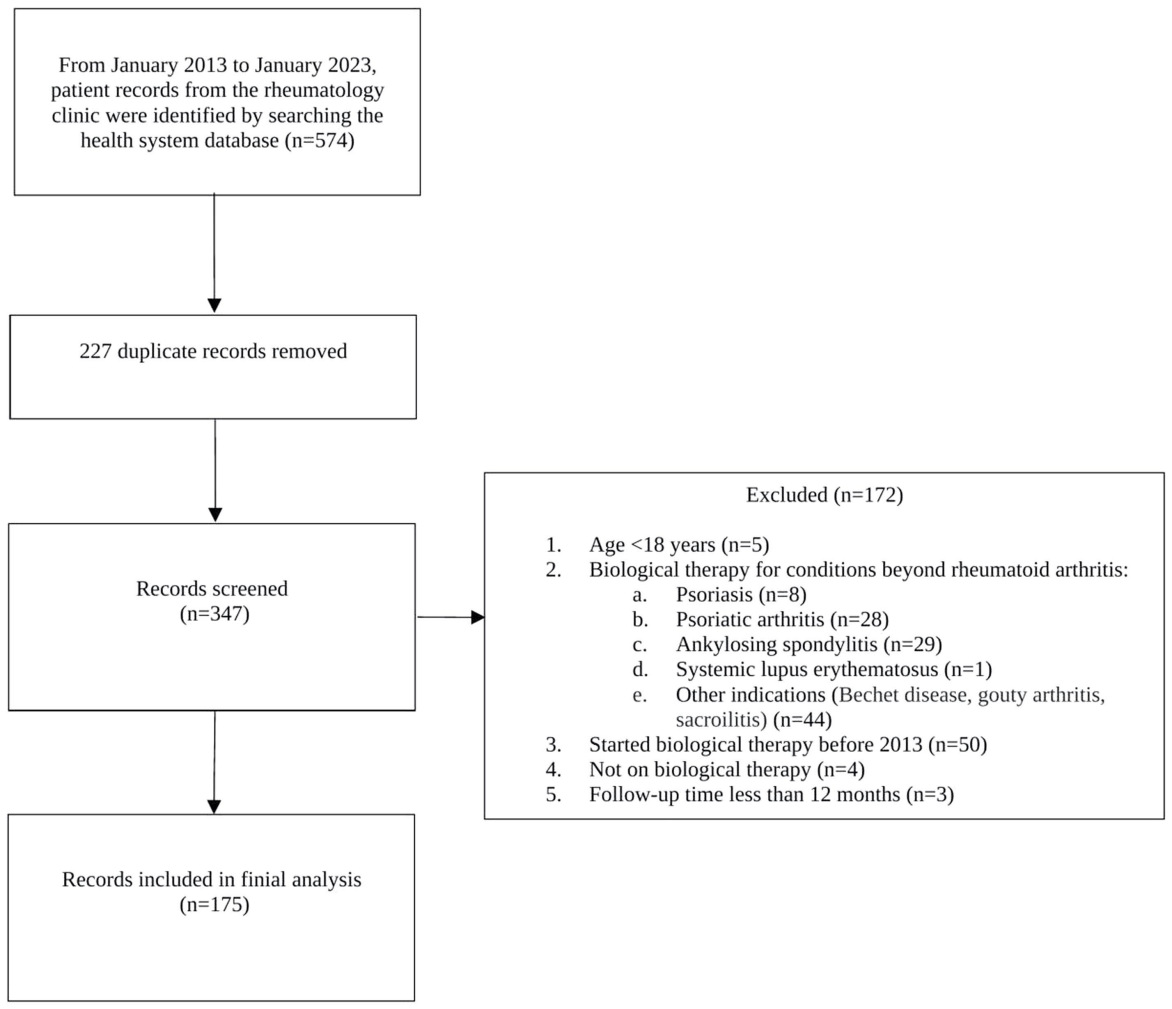
Patients’ selection flowchart.

Of the 347 patient charts evaluated, 175 patients met the inclusion criteria and were included in the analysis (Figure 1). Patients were grouped according to the class of their first (index) therapy: TNFIs (etanercept, adalimumab, and infliximab), non-TNFIs (abatacept, rituximab, and tocilizumab), and tsDMARDs (tofacitinib) (Table 1).

**Table 1:** Baseline characteristics.

Variable	Total (*n* = 175)	TNFIs (*n* = 109)	Non-TNFIs (*n* = 47)	tsDMARDs; tofacitinib (*n* = 19)	*P*-value
Age (median [IQR])	47 [36, 55]	47 [34, 54]	50 [39.50, 56.50]	45 [40.50, 54]	0.316
BMI (kg/m^2^), median (IQR)	27.50 [24.50, 31.30]	27.70 [24.20, 31.30]	27.30 [25.20, 31.20]	27.50 [25.20, 35.20]	0.851
Disease duration year (median [IQR])	9 [6, 12]	9 [6, 12.25]	7 [5, 11]	7 [6.50, 7.50]	0.665
Female, *n* (%)	139 (79%)	82 (75.2)	42 (89.4)	15 (78.9)	0.134
Concomitant comorbidity, *n* (%)					
No comorbid condition	107 (61.1)	69 (63.3)	26 (55.3)	12 (63.2)	0.632
Diabetes mellitus	31 (17.7)	16 (14.7)	13 (27.7)	2 (10.5)	0.103
Hypertension	24 (13.7)	11 (10)	11 (23.4)	2 (10.5)	0.078
Dyslipidemia	5 (2.9)	4 (3.6)	1 (2.1)	0	0.635
Prior stroke	1 (0.6)	1 (0.9)	0	0	0.737
Osteoarthritis	3 (1.7)	2 (1.8)	1 (2.1)	0	0.823
Osteoporosis	2 (1.1)	1 (0.9)	1 (2.1)	0	0.715
Concomitant medications during initial therapy, *n* (%)					
Methotrexate oral	136 (77.7)	82 (75.2)	37 (78.7)	17 (89.5)	0.380
Hydroxychloroquine	92 (52.6)	56 (51.4)	27 (57.4)	9 (47.4)	0.699
Sulfasalazine	7 (4)	3 (2.8)	4 (8.5)	0 (0.0)	0.155
Glucocorticoids	60 (34.7)	39 (36.4)	17 (36.2)	4 (21.1)	0.417
Methotrexate plus hydroxychloroquine	136 (77.7)	82 (75.2)	37 (78.7)	17 (89.5)	0.380
Methotrexate dose in mg (median [IQR])	15 [14.38, 20]	15 [15, 20]	15 [11.88, 17.50]	20 [20, 20]	0.128
RF, *n* (%)^*^	61 (34.9)	37 (33.9)	17 (36.2)	7 (36.8)	NA
TB, *n* (%)^*^	8 (4.6)	3 (2.8)	4 (8.5)	1 (5.3)	0.133
HBV, *n* (%)^*^	1 (0.6)	0	(2.1)	0	0.090

BMI, body mass index. IQR, inter-quartile range; HBV, Hepatitis B virus infection; RF, rheumatoid factor; TB, tuberculosis; TNFIs, tumor necrosis factor inhibitors; non-TNFIs, non-tumor necrosis factor inhibitors; tsDMARDs, targeted synthetic disease-modifying antirheumatic drugs. *Missing data for total sample: RF *n* (%) = 106 (60.6). Anti-Citrullinated Peptide Antibody (ACPA) status was not collected in this study and therefore not reported. Other missing data: TB *n* = 150 (86.7%), HBV *n* = 163 (93.1%).

Baseline characteristics are presented in Table 1, stratified by index therapy class (TNFIs, non-TNFIs, and tsDMARDs). The median (IQR) age of all patients was 47 (36–55) years, with no significant differences across groups (*p* = 0.316). Females comprised 79% of the sample overall, with a slightly higher proportion in the non-TNFI group (89.4%) than in the TNFI (75.2%) and tsDMARDs (78.9%) groups (*p* = 0.134). The median (IQR) disease duration was 9 (6–12) years, with no significant difference across therapy classes (*p* = 0.665).

The most common comorbidities were diabetes mellitus (17.7%), hypertension (13.7%), and dyslipidemia (2.9%), with a numerically higher prevalence of diabetes and hypertension in the non-TNFI group (27.7 and 23.4%, respectively) but no statistically significant differences (*p* = 0.103 and *p* = 0.078, respectively). Overall, 136 patients (77.7%) received methotrexate as concomitant therapy, with a median oral dose of 15 mg (IQR 14.4–20); methotrexate use was highest in the tsDMARDs group (89.5%) but did not differ significantly across groups (*p* = 0.380). Hydroxychloroquine was the second most commonly used concomitant csDMARD, prescribed in 52.6% of patients (*p* = 0.699 across groups). The combination of methotrexate and hydroxychloroquine was used in 77.7% of patients. Rheumatoid factor was positive in 61 patients (34.9%), and missing data were present in 60.6% of the sample.

**Figure 2:**
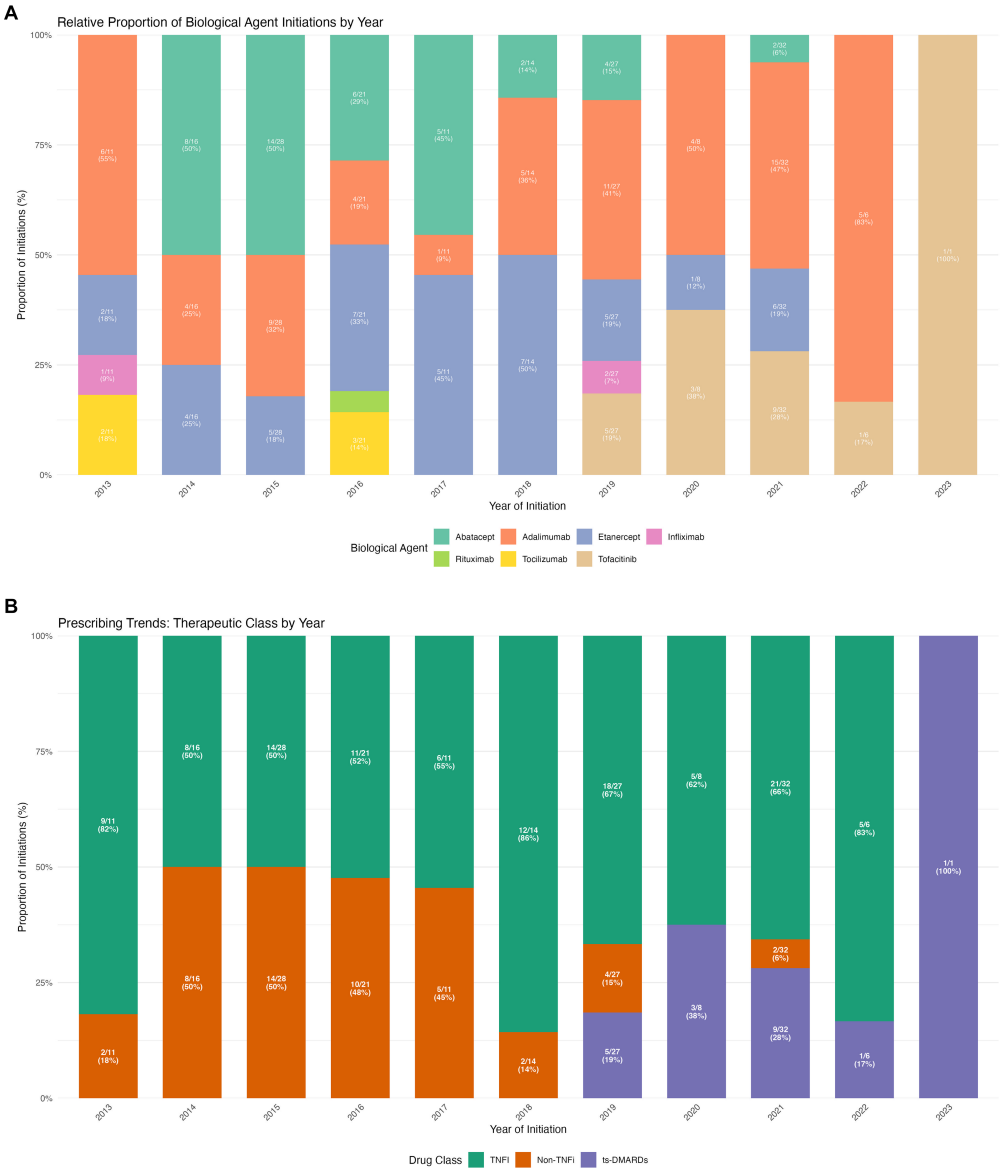
Panel **(A)** shows a stack bar chart that illustrates prescribing trends between 2013 and 2023 in rheumatoid arthritis patients. Panel **(B)** shows a stacked bar illustrating prescribing trends by agent class in rheumatoid arthritis patients.

Figures 2A,B display the annual frequency of initiation of bDMARDs and tofacitinib among the 175 RA patients included in the study from 2013 to 2023. TNFIs were the most frequently initiated class across the entire period. Adalimumab accounted for the highest number of initiations overall, with prominent peaks in 2013 and again during 2019–2022, and a clear reduction in 2017. Etanercept initiations began in 2013 and increased progressively, peaking in 2016 and 2018. Infliximab and non-TNFIs (abatacept, rituximab, tocilizumab) were initiated less often and showed greater year-to-year variability. Tofacitinib had the lowest initiation frequency, accounting for only 10.9% of all first-line advanced therapy initiations in the cohort; its use was negligible before 2019 and occurred primarily between 2019 and 2023, with modest peaks in those years.

### Persistence, switching, and discontinuation patterns

**Figure 3:**
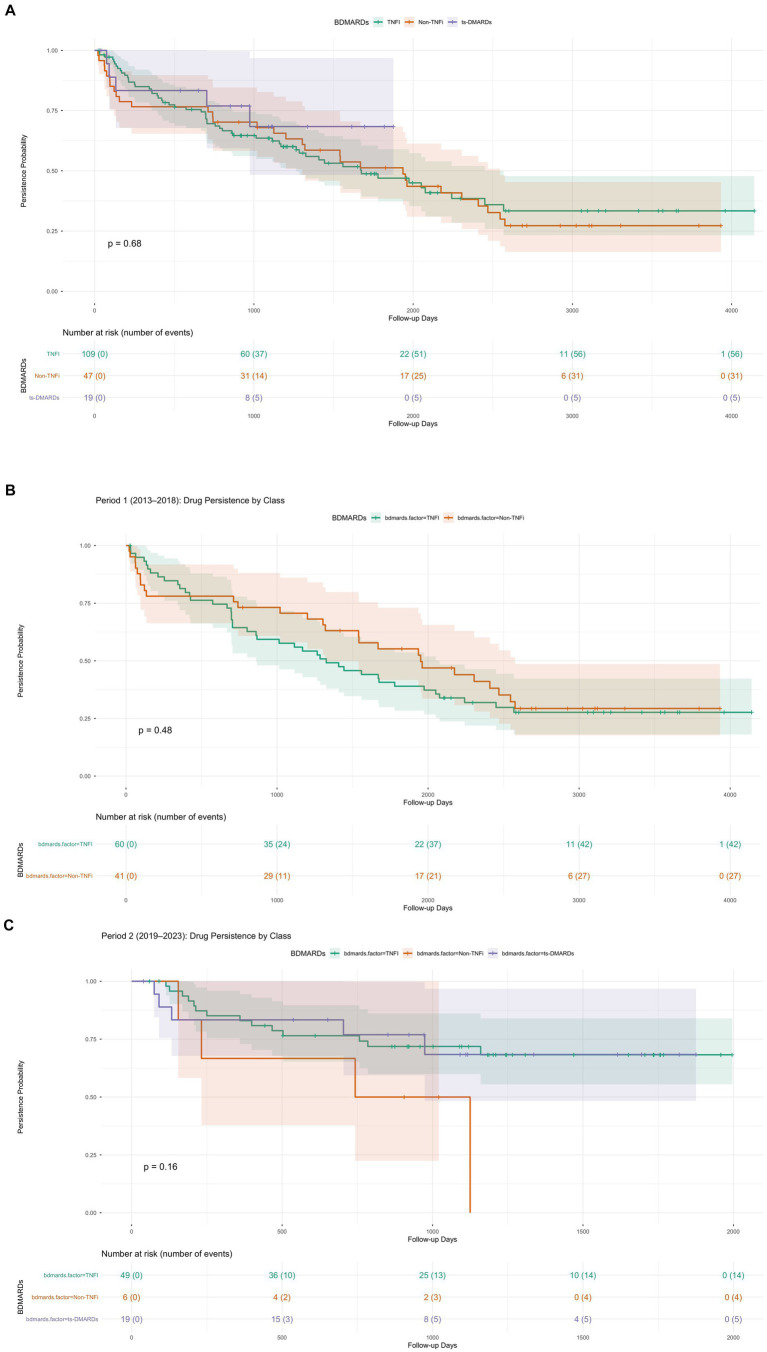
**(A)** Kaplan–Meier survival curves showing treatment persistence on index therapy by therapy class. Confidence intervals are shown by shaded areas. **(B)** Kaplan–Meier survival curves showing treatment persistence on index therapy by therapy class in the period between 2013 and 2018. **(C)** Kaplan–Meier survival curves showing treatment persistence on index therapy by therapy class in the period between 2019 and 2023.

The Kaplan–Meier survival analysis (Figure 3A) assessed treatment persistence on the index therapy (continuous use without discontinuation or switching) over follow-up time (in days), stratified by therapy class: TNFIs, non-TNFIs, and a tsDMARD (tofacitinib). Patients were followed for up to 10 years for TNFIs and non-TNFIs and for up to 5 years for tofacitinib due to its delayed addition to the hospital’s formulary. The log-rank test yielded a *p*-value of 0.68. In the first year after initiation, the estimated probability of persistence (95% CI) was 0.821 (0.751–0.897) for TNFIs, 0.766 (0.654–0.897) for non-TNFIs, and 0.833 (0.678–1.000) for tofacitinib. At 5 years, the corresponding probabilities were 0.470 (0.374–0.590) for TNFIs, 0.513 (0.384–0.684) for non-TNFIs, and 0.684 (0.483–0.968) for tofacitinib. At 10 years, drug survival was 0.334 (0.233–0.478) for TNFIs and 0.272 (0.163–0.454) for non-TNFIs (see Supplementary Table S1 for detailed estimates). The numerically higher persistence observed for tofacitinib in the later years is limited by its shorter maximum follow-up and the small number of patients at risk beyond approximately 2000 days. The overall overlapping curves show comparable treatment retention across the therapy classes in this cohort. In addition, the analysis was stratified by two periods to account for temporal bias and changes in clinical practice. Figures 3B,C show no statistical significance difference between persistence of class agents initiated between 2013 and 2018 (log rank test *p*-value = 0.48) and persistence of class agents initiated between 2019 and 2023 (log rank test *p*-value = 0.16).

The hazard ratios (HRs) for switching or discontinuing bDMARDs and tofacitinib were estimated using univariate and multivariate Cox regression models. In the univariate models, when compared to the TNFI group, the risk of switching or discontinuation was not significantly higher for the non-TNFIs (HR = 1.06, 95% CI: 0.68–1.65, *p* = 0.799) and lower in the tofacitinib group (HR = 0.69, 95% CI: 0.28–1.73, *p* = 0.430). In the multivariable Cox proportional hazards analysis, adjusted for baseline demographic and clinical characteristics, patients in the non-TNFIs group had a non-significantly higher risk of switching or discontinuing treatment than those in the TNFI group (HR = 1.10, 95% CI: 0.69–1.74, *p* = 0.681). However, it was non-significantly lower in the tofacitinib group (HR = 0.68, 95% CI: 0.27–1.72, *p* = 0.420) than in the TNFI group (Table 2).

**Table 2:** Cox proportional hazard models of baseline variables predictive switching or discontinuation of second-line targeted treatment.

Unadjusted Cox proportional hazards model results for bDMARDs^*^			
Variable	Hazard ratio	95% CI	*P*-value
Non-TNFI^§^	1.06	(0.68, 1.65)	0.799
tsDMARDs^§^	0.69	(0.28, 1.73)	0.4301
Adjusted Model Cox proportional hazards model results for bDMARDs^‡^			
Non-TNFI^§^	1.10	(0.69, 1.74)	0.681
tsDMARDs^§^	0.68	(0.27, 1.72)	0.420
BMI	1.00	(0.99, 1.00)	0.768
Age	0.99	(0.97, 1.00)	0.208
Diabetes mellitus	0.75	(0.41, 1.37)	0.353
Steroid therapy	1.11	(0.70, 1.70)	0.646
Methotrexate	1.28	(0.74, 2.20)	0.378

^§^References group is TNFI.

^*^Undjusted Cox Proportional Global Schoenfeld Test: Global Schoenfeld Test: *p* = 0.6715, AIC: 841.95, Concordance Index: 0.52.

^‡^Adjusted Global Schoenfeld Test: *p* = 0.3749, Individual Schoenfeld Test *p*-values: bDMARDs: 0.6947, BMI: 0.1333, Age: 0.203, Diabetes mellitus: 0.5155, Steroids use: 0.4211, Methotrexate use: 0.8437, GLOBAL: 0.3749, AIC: 822.98, Concordance Index: 0.57.

TNFIs, tumor necrosis factor inhibitors; non-TNFIs, non-tumor necrosis factor inhibitors; tsDMARDs, targeted synthetic disease-modifying antirheumatic drugs; BMI, Body mass index.

**Figure 4:**
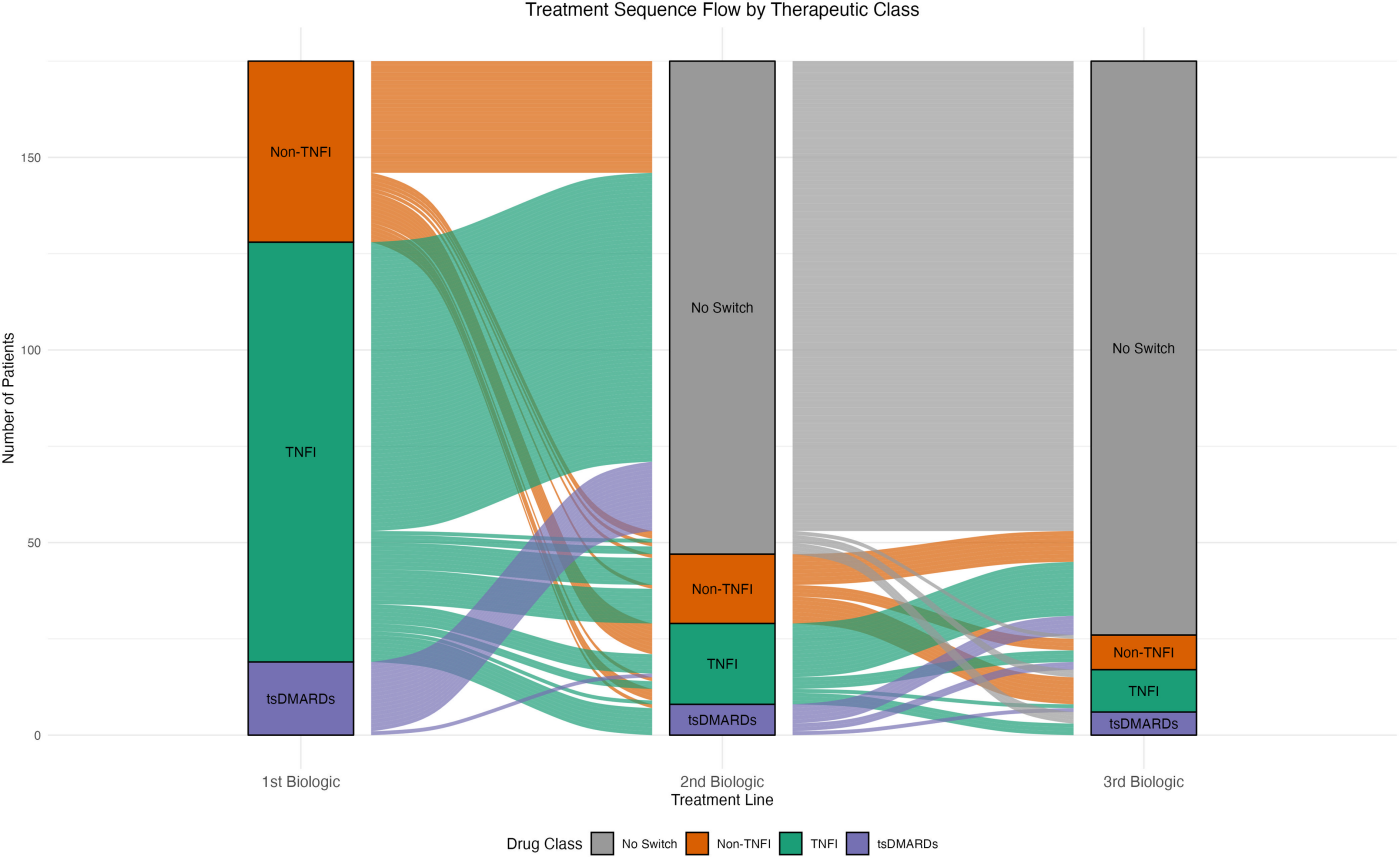
Biological agents treatment pattern flowchart: The graph depicts the transition of patients from their initial biological agent class to a second or third biological agent class, with threads extending from the bars. Patients who did not switch agents are represented by the No Switch bar. Some patients experienced a discontinuity in their second biological and subsequently returned to the same biological (6 cases), which is illustrated by threads emanating from the not-switched second biological agent bar. Bar height indicates patient count per class at each line. Threads show individual patient transitions between treatment lines. ‘No switch’ = patient did not advance to the next treatment line. TNFIs (Adalimumab, Etanercept, Infliximab); non-TNFIs (abatacept, tocilizumab, rituximab); tsDMARDs (tofacitinib).

The treatment patterns and transitions between bDMARDs and tofacitinib are illustrated in Figure 4, a Sankey diagram depicting the flow of therapy use across up to three lines. Among the 175 cases included in the analysis, 73.1% (128 cases) remained on their initial biological agent without switching, 22.3% (39 cases) switched to a different class, and 4.6% (8 cases) switched within the same class. Among the 47 patients who switched to a second biological agent, 57.4% (27 cases) persisted on that second agent, 34.0% (16 cases) switched to a different class, and 8.5% (4 cases) switched within the same class.

Among the 128 patients (73.1%) who persisted on their initial biological agent, adalimumab was the most common agent associated with drug survival (44 patients, 34.4%), followed by etanercept (33 patients, 25.9%), abatacept (29 patients, 22.7%), tofacitinib (18 patients, 14.1%), tocilizumab (2 patients, 1.6%), and infliximab and rituximab (1 patient each, 0.8%). In the 47 cases (27%) who experienced switching to a second agent, the most frequent transitions were from adalimumab to abatacept (8 patients, 17%) and from abatacept to adalimumab (6 patients, 12.8%) (see Figure 4 and Supplementary Table S2 for details).

At the second follow-up (third-line therapy), 27 of 47 patients (57.4%) remained on their second biological agent. Drug survival was highest with adalimumab (9 cases, 19.2%), followed by etanercept (5 cases, 10.6%), tofacitinib (5 cases, 10.6%), abatacept (4 cases, 8.5%), and tocilizumab (4 cases, 8.5%). Additionally, 20 of the 47 cases (42.6%) experienced further switching, with the most common transitions being from abatacept to etanercept (4 cases, 20%), etanercept to tofacitinib (2 cases, 10%), and tofacitinib to tocilizumab (2 cases, 10%) (Figure 4 Sankey for therapeutic class; Supplementary Figure S1 for granular view in the supplement; and Supplementary Table S2).

The reasons for discontinuation and switching of bDMARDs and tofacitinib during the study period are summarized as follows. Among the 31 patients who discontinued TNFIs, the most common reasons were pregnancy (6 patients, 19.4%), adverse drug events (4 patients, 12.9%), non-adherence (3 patients, 9.7%), and infection (3 patients, 9.7%), with lack of efficacy reported in only 1 patient (3.2%); reasons were not reported in 10 cases (32.3%). For the 19 patients who discontinued non-TNFIs, non-adherence was the leading cause (4 patients, 21.1%), followed by pregnancy (2 patients, 10.5%), lack of efficacy (3 patients, 15.8%), and adverse drug events (1 patient, 5.3%), with no reported reasons in 7 cases (36.8%). Among the 5 patients who discontinued tsDMARDs (tofacitinib), non-adherence (2 patients, 40%) and pregnancy (1 patient, 20%) were noted, with no reported reasons in 2 cases (40%).

Among the 40 patients who switched from TNFIs, lack of efficacy was the predominant reason (24/40, 60%). Given that all patients were b/tsDMARD-naïve at initiation of index therapy, these cases predominantly represent primary non-response, although a minority may reflect secondary loss of response. We were unable to formally distinguish between the two due to the retrospective nature of the data and the lack of standardized longitudinal documentation of disease activity. This was followed by adverse drug events (6 patients, 15%), issues related to injection formulation (2 patients, 5%), and unavailability (1 patient, 2.5%), with no reported reasons in 7 cases (17.5%). Among the 28 patients who switched from non-TNFIs, lack of efficacy was the most frequent reason (21 patients, 75%), followed by adverse drug events (4 patients, 14.3%) and pregnancy (1 patient, 3.6%), with no reported reasons in 2 cases (7.1%). Of the 6 patients who switched from tsDMARDs, lack of efficacy was cited in 3 cases (50%), non-adherence in 1 (16.7%), and no reason was reported in 2 cases (33.3%). Specific adverse events are included in Table 3.

**Table 3:** Reasons for discontinuation and switching of biological agents during the study period.

Reasons of discontinuation no, (%)	TNFIs (Total *N* = 31)	Non-TNFIs (Total *N* = 19)	tsDMARDs(Total *N* = 5)
Lack of efficacy	1 (3.2)	3 (15.8)	–
Adverse drug event^*^	4 (12.9)	1 (5.3)	–
Pregnancy	6 (19.4)	2 (10.5)	1 (20)
Unavailability	1 (3.2)	–	–
Non-adherence	3 (9.7)	4 (21.1)	2 (40)
Infection^^^	3 (9.7)	–	–
Other illnesses^¤^	–	1 (5.3)	–
Death^&^	1 (3.2)	–	–
Remission^°^	2 (6.5)	–	–
Patient preference to switch to an oral formulation	–	1 (5.3)	–
Not reported	10 (32.3)	7 (36.8)	2 (40)

Reasons of switching no, (%)	TNFIs (Total *N* = 40)	Non-TNFIs (Total *N* = 28)	tsDMARDs(Total *N* = 6)
Lack of efficacy	24 (60)	21 (75)	3 (50)
Adverse drug event^**^	6 (15)	4 (14.3)	–
Pregnancy	–	1 (3.6)	–
unavailability	1 (2.5)	–	
Non-adherence	–	–	1 (16.7)
Issue related to the formulation^†^	2 (5)	–	–
Not reported	7 (17.5)	2 (7.1)	2 (33.3)

*Adverse effect (n): Elevated liver enzyme with abatacept (1), skin abscess with adalimumab (1), laryngeal nodule with etanercept (1), interstitial lung disease (ILD) with adalimumab (1), skin and malar rash suspected TNF inhibitor-induced lupus with etanercept (1).

**Adverse effect (n): Rash, multiple scaly erythmatous lesion with adalimumab (1), cardiomyopathy with adalimumab (1), Allergic reaction with Infliximab (1), and adalimumab (2), Elevated liver enzyme with etanercept (1), Hypersenstity reaction with tocilizumab (3), leukopenia and thrompocytopenia with tocilizumab (1).

^†^Difficulty in adminstration the injection with etanercept (2).

^¤^Other illness (n): New breast cancer diagnosis and started on chemotherapy (1).

^Infection (n): Addressing latent TB on patient started on etanercept (1), HBV activation with adalimumab (1), Active TB (1) with etanercept (1).

^&^Death (n): Urelated secondary to complicated perforated ileum on etanercept.

°Remission (n): with adalimumab (1) and etanercept (1).

## Discussion

Our retrospective cohort study provides initial real-world insights from a single tertiary center (DMC) in the Eastern Province of Saudi Arabia into persistence, switching, and discontinuation patterns of b/tsDMARDs among biologic-naïve patients with RA.

Over the 10-year period (2013–2023), TNFIs were the most commonly initiated first-line advanced therapy (62.3%), followed by non-TNFIs (26.9%) and tofacitinib (10.9%). This predominance of TNFIs aligns with global patterns observed in Europe, Japan, the United States, and other regions, where TNFIs have remained the preferred first-line option due to established efficacy, long-term safety data, and physician familiarity.

Similar prescribing preferences for TNFIs as first-line agents have been observed in other real-world cohorts worldwide. For instance, a nationwide retrospective cohort study conducted in Korea showed that the most common initial treatment regimen was adalimumab + methotrexate (98/614, 16.0%), followed by etanercept + methotrexate (72/614, 11.7%) and tocilizumab + methotrexate (68/614, 11.1%) (13). Another cohort study of 8,027 adult patients with RA enrolled in the Corrona RA registry in the United States reported that csDMARD monotherapy and TNFI + csDMARD combination therapy were the most common index therapies, with usage rates of 39.9 and 44.9%, respectively during 2004–2007; 38.6 and 38.2%, respectively, during 2008–2011; and 35.2% for both during 2012–2015 (14). Another study from Taiwan found that a majority of patients (70.0%) used TNFIs as their index treatment between 2012 and 2017 (11). Moreover, a nationwide, population-based, retrospective cohort analysis of 5449 patients with RA found that 4202 received TNFIs and 1247 received tocilizumab between January 2014 and December 2017 (15). In addition, a U.S. claims-based analysis by Huang et al. examined 3,174 commercially insured adult patients who initiated methotrexate (MTX)-based combination therapy between 2012 and 2013. In this cohort, approximately 49% added a csDMARD to MTX, 42% added a TNFI, 8% added a non-TNFI, and 1% added a tsDMARD (16). Similarly, a study by Rosenberg et al., analyzed 753 adult RA patients (61.8% treatment-naïve) found that the most common drug class used was TNFIs [adalimumab, infliximab, golimumab, etanercept, and certolizumab pegol], followed by the JAK inhibitor [tofacitinib], IL-6 inhibitors [tocilizumab and sarilumab], anti-CD20 [rituximab], and CTLA4-Ig [abatacept], respectively among treatment-naïve patients initiating first-line therapy (17). Another study from Germany found that TNFIs (47.8%) and JAKIs (29.4%) were most frequently prescribed among 4,678 patients (18).

Due to the low sample size, the Kaplan–Meier analysis lacked a significant difference in treatment persistence across the three therapy classes (log-rank *p* = 0.68), with 5-year survival probabilities of 0.47 for TNFIs, 0.51 for non-TNFIs, and 0.68 for tofacitinib, and 10-year survival of 0.33 and 0.27 for TNFIs and non-TNFIs, respectively. These rates align with international registries and regional cohorts. For instance, a study in the United States involving 705 patients found no difference in persistence over the 9-year study period between abatacept and a TNFI (adalimumab, certolizumab, etanercept, golimumab, or infliximab) when used as a first-line biologic agent (*p* = 0.7406). However, as a second-line biologic agent, patients treated with abatacept had a greater persistence than those treated with a TNFI, based on both univariate and multivariate (controlling for all baseline covariates using the Cox proportional hazard model) analyses (*p* = 0.0001) (19). Moreover, a propensity score-matched analysis of the Australian OPAL dataset (2015–2018) — including 1,300 bDMARD and 650 tofacitinib patients — showed comparable disease activity score (DAS) remission rates at 18 months and similar median treatment persistence: tofacitinib 34.2 months (95% CI 32.2–not reached) vs. bDMARDs 33.8 months (95% CI 28.8–40.4); *p* = 0.19 (log-rank test) (20).

Unlike our study, several observational studies have reported significantly higher persistence rates with non-TNFIs compared to TNFIs. For instance, So et al. (15) found that TNFis (adalimumab, etanercept, infliximab, and golimumab) had significantly lower drug persistence than tocilizumab as first-line biologic therapy in Korean patients with RA (log-rank test, *p* < 0.001). After adjustment for confounders, TNFIs were associated with a higher risk of discontinuation compared with tocilizumab (hazard ratio [HR] 1.63, *p* < 0.001). In subgroup analyses, all TNFIs except infliximab showed significantly lower persistence than tocilizumab (15). Similarly, Hishitani et al. (21) demonstrated that tocilizumab had a higher drug retention rate than infliximab and adalimumab (but not etanercept) in Japanese patients with RA. Ebina et al. (22) reported higher overall retention with abatacept and tocilizumab than with infliximab among 1,037 Japanese patients with RA in the ANSWER cohort. Dos Santos et al. (23) analyzed 66,787 Brazilian patients initiating first-line biologics and found that abatacept had the highest 12-month persistence, followed by golimumab, tocilizumab, etanercept, and adalimumab, with certolizumab pegol and infliximab showing the lowest rates.

Park et al. (24) evaluated 2,684 Korean patients who initiated bDMARDs between 2013 and 2014 and reported the highest persistence with abatacept among initiators (69.3%) and with tocilizumab among switchers (77.0%), while adalimumab had the lowest rates in both groups (48.2 and 28.8%, respectively). Adalimumab and etanercept were significantly more likely to be non-persistent than infliximab among initiators (HR 1.58, 95% CI 1.27–1.96; HR 1.42, 95% CI 1.14–1.76), whereas tocilizumab was associated with significantly higher persistence among switchers (HR 0.411, 95% CI 0.206–0.819) (24). Finally, Stamm et al. (25) assessed drug survival in both bio-naïve and bio-experienced Austrian patients and found the highest survival with tocilizumab and abatacept, and the lowest with infliximab.

Moreover, real-world evidence also highlights differences by treatment line. In a cohort of biologic-naïve and treatment-experienced patients (17), overall drug survival was higher in treatment-naïve patients than in treatment-experienced patients. Among treatment-naïve patients, tocilizumab showed the highest 12-month survival, while golimumab led at 24 and 36 months (with tocilizumab second and etanercept comparable to tocilizumab). Lower survival was observed with tofacitinib, abatacept, and golimumab throughout the follow-up period. In treatment-experienced patients, tocilizumab, etanercept, tofacitinib, abatacept, and golimumab had the highest rates at 12 and 24 months, with similar survival (20–30%) at 36 months for golimumab, tofacitinib, abatacept, and tocilizumab. In both groups, adalimumab, infliximab, and rituximab consistently showed the lowest survival (17).

Additionally, a large Australian nationwide study using the OPAL dataset [approximately 4,500 RA patients; (26)] reported 12-month persistence was highest for upadacitinib (72%), followed by baricitinib (61%), subcutaneous TNFis (58%), tocilizumab (55%), tofacitinib (53%), and abatacept (49%). Persistence improved with combination therapy (JAK inhibitors or other agents plus methotrexate/csDMARDs) versus monotherapy. First-line patterns were similar: 68% for upadacitinib and baricitinib, 55% for tofacitinib, 49% for TNF inhibitors, 55% for abatacept, and 57% for tocilizumab (26). In a German RHADAR database analysis (18), 5-year drug survival was highest for JAKIs (68.3%), followed by TNFIs (58.6%), IL-6Is (58.6%), abatacept (55.0%), and rituximab (53.3%). Adjusted Cox regression showed higher discontinuation risk versus JAKIs for rituximab (HR 1.36, *p* = 0.015), abatacept (HR 1.46, *p* < 0.001), IL-6Is (HR 1.20, *p* = 0.026), and TNFIs (HR 1.29, *p* < 0.001) (18).

Taken together, real-world evidence on the persistence of b/tsDMARDs in RA demonstrates substantial variability, influenced by factors such as treatment line (first-line vs. later lines), patient population (biologic-naïve vs. experienced), geographic region, and the specific drugs compared.

While we did not examine the factors influencing prescribing patterns, previous studies have investigated them to gain a better understanding of current clinical practices (27–29). Erkan et al. (27) discovered that physicians’ preferences and experience, as well as medication costs, play a significant role in influencing treatment choices. In the 2020 study by Sullivan et al. (28) on b/tsDMARD prescription patterns for RA among US physicians, the top reasons for choosing the first targeted therapy were focused on efficacy and disease control. Physicians most commonly cited strong overall efficacy (79.9%), followed by disease progression inhibition (60.2%) and reduction in stiffness (58.5%). These factors drove the dominance of TNF inhibitors as first-line options, with other considerations, such as maintenance of efficacy, steroid reduction, or delivery method, varying by therapy class but ranking lower overall (28). Moreover, in a 2020 study by Sullivan et al. (29) on b/tsDMARDs prescription patterns among rheumatologists in Europe (EU5: France, Germany, Italy, Spain, UK) and Japan, physicians prioritized strong overall efficacy as the top reason for choosing the first targeted therapy, cited by 75.3% in EU5 and 77.3% in Japan. In EU5, this was followed by inhibition of disease progression (46.4%) and a good overall safety profile (40.0%). In Japan, the next most common reasons were fast onset of action (53.8%) and inhibition of disease progression (55.5%). These efficacy-driven factors contributed to TNFI dominance as first-line therapy (80.8% in EU5, 64.6% in Japan), with safety and rapid action showing regional variations (29). Other studies (30, 31) highlighted the importance of patient preferences when selecting among different biologic agents, including factors such as the route of administration, frequency, dosage form, and the individual’s past experiences and any adverse reactions to the drug.

For patients with RA not achieving the treatment target on a b/tsDMARD, the current study demonstrated that switching between different drug classes was more common than switching within the same drug class, which aligns with the 2021 ACR guideline’s conditional recommendation favoring switching to an agent of a different class over same-class cycling (based on very low-certainty evidence suggesting potential benefits in improving disease activity and extending drug survival) (8), even though the 2022 EULAR recommendations permit switching to any other bDMARD (same or different class) or tsDMARD without a conditional preference for a different mechanism of action while weighing specific safety risks (9). Reasons for discontinuation were similar to those previously reported for DMARDs (21, 22, 29). However, other reasons for switching/discontinuation not mentioned in our study include patient preference, change in hospital (22).

This study has several limitations. First, the cohort was restricted to b/tsDMARD-naïve patients initiating first-line advanced therapy, excluding treatment-experienced individuals. This design limits generalizability to the broader RA population in routine practice, where many patients require second- or later-line therapies due to inefficacy, adverse events, or disease progression. As a result, the analysis could not evaluate how prior treatment exposure influences drug survival, switching patterns, or class-specific outcomes, key aspects frequently reported in real-world studies that stratify by treatment line. To address potential temporal bias arising from changes in clinical practice over the 10-year study period, a two-period analysis was performed (2013–2018 and 2019–2023). The current analysis showed no statistically significant difference in the persistence of agents initiated during the same period. Second, a very important consideration of the current analysis is the small sample size (n = 175), which can reduce statistical power, particularly for subgroup analyses (e.g., only 19 patients initiated tofacitinib) and long-term outcomes. Although the current analysis may be viewed as descriptive, statistical models can be the best descriptive tools, even if not intended for direct inference (32). In addition, the goal of analysis in observational study designs using existing databases is not to detect significance (which is a binary statement about whether an effect exists), but rather to provide unbiased estimates (33). In the current study, we provide estimates of persistence probabilities in Supplementary Table S1. Although the sample size is small, and the resulting estimates may be imprecise, the publication of observational studies should not be discouraged on this basis alone. Rather, publishing observational studies even with imprecise estimates can contribute to the cumulative evidence base; as more studies become available, their estimates can be pooled through meta-analysis to generate more precise summary estimates (33).

Third, substantial missing data on key prognostic factors limited the robustness of adjustment for seropositivity, baseline disease severity, or joint damage. RF status was available for only 39.4% of the cohort (missing in 60.6%), while ACPA data were completely absent, as ACPA is not routinely collected in DMC. Serial disease activity scores (e.g., DAS28) were also unavailable. This level of incompleteness is common in retrospective real-world studies relying on routine clinical records from hybrid paper-electronic systems, particularly during Saudi Arabia’s progressive EHR implementation under Vision 2030 (2013–2023). Serological results may not have been systematically re-documented if previously known, performed externally, or not repeated when they did not alter management. In addition, many real-world datasets derived from administrative or treatment records do not routinely link structured laboratory data.

Although high-titer RF and/or ACPA are consistently associated with erosive disease and poorer long-term outcomes in RA, their utility in predicting differential response to specific b/tsDMARDs is more limited (9, 34). A recent meta-analysis of randomized controlled trials showed that, in csDMARD-naïve and csDMARD-inadequate-responder patients, seropositivity was not associated with a better response to bDMARDs (pooled 6-month ACR20 relative risk ratios ≈of 1.02–1.09), with generally comparable efficacy between seropositive and seronegative patients across mechanisms of action. Only in TNFi-experienced patients was there some evidence of greater benefit in seropositive individuals. These findings suggest that the absence of comprehensive RF/ACPA data in the present study is unlikely to have substantially biased the observed class-level persistence patterns (34).

We addressed this limitation by summarizing missing data descriptively and excluding RF and ACPA from the multivariable Cox models. Nevertheless, the lack of comprehensive serological profiling limits serostatus-stratified subgroup analyses and may affect the generalizability of our findings to populations in which seropositivity strongly influences prognosis.

Fourth, because this was a single-center study, patients who transferred to other facilities were censored at their last visit; we cannot rule out subsequent treatment changes at external centers.

Moreover, the inability to differentiate primary from secondary failure for lack-of-efficacy events is a limitation inherent to the retrospective design and the absence of systematic DAS28 or CDAI assessments.

Finally, other unmeasured confounders—including patient adherence, socioeconomic factors, detailed safety-monitoring practices, and reasons for treatment discontinuation or switching—may have influenced the observed patterns. Despite these constraints, the study provides valuable foundational real-world evidence on first-line b/tsDMARD use in a Saudi RA cohort, addressing an important evidence gap in the Middle East. Future multicenter studies with standardized, prospective data collection are warranted.

## Conclusion

In conclusion, TNFIs remain the cornerstone of first-line advanced RA therapy in this Saudi cohort, with comparable persistence across b/tsDMARD classes in routine practice. The numerically favorable survival for tofacitinib warrants further exploration in adequately powered multi-center studies. Future research should incorporate multi-center designs, disease activity metrics (e.g., DAS28), patient-reported outcomes, and economic analyses to better inform personalized treatment strategies and optimize resource allocation in Saudi Arabia’s evolving rheumatology landscape.

## Data Availability

The original contributions presented in the study are included in the article/Supplementary material, further inquiries can be directed to the corresponding author.

## Supplementary material

SUPPLEMENTARY FIGURE S1 Illustrates the sequence and flow of biological and targeted synthetic DMARDs (b/tsDMARDs) used by the study cohort.
