# Supplementary material for: Persistence, switching, and discontinuation patterns of b/tsDMARDs in patients with rheumatoid arthritis: a single-center retrospective cohort study from Saudi Arabia
Source: Front Med (Lausanne). 2026 Jul 24;13:1855256. doi: 10.3389/fmed.2026.1855256 (PMC13447146; doi:10.3389/fmed.2026.1855256)
Supplement: Supplementary file 1 [file Table_1.DOCX]

**Supplementary Materials**

**Table S1: Biologic and targeted synthetic DMARDs Survival Rates**

| **Year** | **Patients at risk** | **Event** | **Survival** | **95% CI** |
| --- | --- | --- | --- | --- |
| **Tumor necrosis factor inhibitors (TNFIs)** | | | | |
| 1 | 87 | 19 | 0.821 | (0.751, 0.897) |
| 2 | 71 | 13 | 0.696 | (0.613, 0.790) |
| 3 | 56 | 6 | 0.636 | (0.549,0.735) |
| 4 | 38 | 8 | 0.532 | (0.439, 0.644) |
| 5 | 26 | 4 | 0.470 | (0.374, 0.590) |
| 6 | 17 | 3 | 0.409 | (0.310, 0.539) |
| 7 | 14 | 2 | 0.359 | (0.258, 0.500) |
| 8 | 11 | 1 | 0.334 | (0.233, 0.478) |
| 9 | 7 | 0 | 0.334 | (0.233, 0.478) |
| 10 | 4 | 0 | 0.334 | (0.233, 0.478) |
| **Non-tumor necrosis factor inhibitors (Non-TNFIs)** | | | | |
| 1 | 36 | 11 | 0.766 | (0.654, 0.897) |
| 2 | 35 | 1 | 0.745 | (0.630, 0.880) |
| 3 | 29 | 3 | 0.679 | (0.558, 0.827) |
| 4 | 24 | 4 | 0.586 | (0.458, 0.748) |
| 5 | 21 | 3 | 0.513 | (0.384, 0.684) |
| 6 | 15 | 4 | 0.408 | (0.284, 0.588) |
| 7 | 11 | 4 | 0.300 | (0.186, 0.482) |
| 8 | 7 | 1 | 0.272 | (0.163, 0.454) |
| 9 | 3 | 0 | 0.272 | (0.163, 0.454) |
| 10 | 2 | 0 | 0.272 | (0.163, 0.454) |
| **Targeted synthetic disease-modifying antirheumatic drug (ts-DMARDs); tofacitinib** | | | | |
| 1 | 15 | 3 | 0.833 | (0.678, 1.000) |
| 2 | 12 | 1 | 0.769 | (0.593, 0.997) |
| 3 | 7 | 1 | 0.684 | (0.483, 0.968) |
| 4 | 4 | 0 | 0.684 | (0.483, 0.968) |
| 5 | 1 | 0 | 0.684 | (0.483, 0.968) |

**Table S2: Patterns of Biological Drug Use: Counts and Percentages**

| **Treatment Sequence / Pattern** | **Count/ total count (%)** |
| --- | --- |
| **Persistence on first-line therapy (no switch)** | **128/175 (73.0)** |
| Adalimumab | 44 (34.4) |
| Etanercept | 33 (25.9) |
| Abatacept | 29 (22.7) |
| Tocilizumab | 2 (1.6) |
| Tofacitinib | 18 (14.1) |
| Infliximab | 1 (0.8) |
| Rituximab | 1 (0.8) |
| **Total switch events to second-line** | **47/175 (27.0)** |
| **Switch between classes** | **39/ 47 (83.0 )** |
| Adalimumab to abatacept | 8 (17.0) |
| Abatacept to adalimumab | 6 (12.8) |
| Adalimumab to tocilizumab | 5 (10.6) |
| Adalimumab to tofacitinib | 4 (8.5) |
| Abatacept to etanercept | 3 (6.4) |
| Etanercept to tofacitinib | 3 (6.4) |
| Tocilizumab to etanercept | 2 (4.3) |
| Abatacept to tocilizumab | 2 (4.3) |
| Abatacept to tofacitinib | 1 (2.1) |
| Etanercept to abatacept | 1 (2.1) |
| Etanercept to tocilizumab | 1 (2.1) |
| Infliximab to abatacept | 1 (2.1) |
| Tocilizumab to adalimumab | 1 (2.1) |
| Tofacitinib to adalimumab | 1 (2.1) |
| **Switches within class** | **8/47 (17.0)** |
| Etanercept to adalimumab | 4 (8.5) |
| Adalimumab to etanercept | 3 (6.9) |
| Infliximab to adalimumab | 1 (2.1) |
| ***Second-line to third-line therapy*** | ***47/47 (100.00)*** |
| **Persistence on second-line therapy (no switch)** | **27/47 (57.45)** |
| Adalimumab | 9 (19.15) |
| Etanercept | 5 (10.64) |
| Tofacitinib | 5 (10.64) |
| Abatacept | 4 (8.51) |
| Tocilizumab | 4 (8.51) |
| **Total switch events to third-line** | **20/47 (42.55)** |
| **Switches between classes** | **16/20 (80.0)** |
| Abatacept to etanercept | 4 (20.0) |
| Etanercept to tofacitinib | 2 (10.0) |
| Tofacitinib to tocilizumab | 2 (10.0) |
| Adalimumab to abatacept | 2 (10.0) |
| Adalimumab to tofacitinib | 1 (5.0) |
| Etanercept to tocilizumab | 1 (5.0) |
| Tocilizumab to adalimumab | 1 (5.0) |
| Tocilizumab to etanercept | 1 (5.0) |
| Tocilizumab to infliximab | 1 (5.0) |
| Tofacitinib to etanercept | 1 (5.0) |
| **Switches within class** | **4/20 (20.0)** |
| Abatacept to tocilizumab | 2 (10.0) |
| Adalimumab to etanercept | 1 (5.0) |
| Tocilizumab to abatacept | 1 (5.0) |
| **Discontinuation with restart of the same biologic** | **6 cases** |
| Restarting tofacitinib | 3 |
| Restarting etanercept | 2 |
| Restarting abatacept | 1 |

^#^Medication interrupt and restart the same biological
